# Orientation of medical trainees to a new clinical environment (the ready-steady-go model): a constructivist grounded theory study

**DOI:** 10.1186/s12909-022-03105-3

**Published:** 2022-01-14

**Authors:** Anél Wiese, Deirdre Bennett

**Affiliations:** Medical Education Unit, School of Medicine, University College Cork, Cork, Ireland

**Keywords:** Postgraduate medical education, Trainee orientation, Faculty development

## Abstract

**Background:**

High-quality orientation of trainees entering a new clinical workplace is essential to support education and patient safety. However, few consultants receive extensive formal training to support new trainees and must create their own ways of integrating newcomers into their clinical team and work environment. We aim to conceptualise the strategies consultants use in the early stages of working with new trainees that will be useful for future faculty development in this area.

**Methods:**

We used constructivist grounded theory (CGT) methodology by interviewing fifteen consultants in three medical specialties, to explore how trainees are integrated into a new clinical environment. We used CGT principles and procedures (iteration, constant comparison, and theoretical sampling) to analyse and construct a conceptual interpretation of the empirical data.

**Results:**

Consultants’ central concern when introduced to a new cohort of trainees was that they had the required knowledge and skills (*ready*), were adapted and integrated into the new workplace and clinical team (*steady*), and safely participating in practice (*go*). Consultants used two broad strategies: formal orientation and informal orientation. Both these approaches had the common goal of intensifying interaction between consultants and trainees to get trainees to a position where they were ready, adapted, integrated, and participating safely and efficiently in practice. Several disruptors were identified by participants that delayed and sometimes completely inhibited the orientation process.

**Conclusions:**

The model of orientation constructed through this research could be a valuable tool to support faculty development initiatives, the reflective learning practice of clinical supervisors, and curriculum design. The disruptors were identified as valid priorities for improving trainee orientation in postgraduate medical education. Future research should involve a longitudinal approach to explore trainee engagement with orientation upon entering a new clinical workplace.

## Background

Postgraduate medical training programmes are designed to allow trainees to rotate through a series of posts at regular intervals [1]. When trainees start working in a new clinical environment, they require supervision and support to adapt to a new set of circumstances, including changes in the scope of practice, specialty, clinical team, clinical supervisors, and service demands [2]. The rotational approach of medical training creates upheaval for trainees and consultants but allows for greater diversity and breadth of clinical experience, teaches trainees how to adapt and cope with multiple practice styles and promotes greater trainee independence [3]. The downside of this approach is that it negatively impacts the continuity of practice and education and disrupts meaningful relationships with patients, supervisors, and peers [3, 4]. Patient safety is the most crucial issue to consider when trainees are introduced to a new clinical environment. Research has shown that rotation transitions and the arrival of new medical trainees can lead to an increase in patient mortality and medication errors [5, 6]. Inadequate supervision has been identified as a contributing factor to medical errors and malpractice claims involving trainees [7, 8]. Consultants and trainees need guidance and support on managing the effects of multiple and frequent rotations, navigating unfamiliar clinical workplaces, and coping with changing responsibilities and expectations.

The trainee perspective of working in an unfamiliar clinical environment has been well-studied but how consultants prepare for and deal with new trainees at the frontline of service delivery is less clear. Many trainees find transition experiences challenging and stressful [9–11]. The process of moving into a new workplace is a critically intensive learning period that is influenced and shaped by the peculiarities of the new setting [12]. Trainees must develop new ways of participating in clinical practice, which may differ from previous working experiences as they take their place in new workplace culture and local practice. Support from consultants could buffer these initial stressors for trainees and facilitate the on-the-job learning required to develop areas of practice relevant to a particular clinical setting. Supervision by consultants promotes safe patient care, workplace learning, and professional development by supporting participation, observation, and dialogue about day-to-day medical practice between consultants and trainees [13]. As in all aspects of postgraduate medical education, consultants likewise play a pivotal part in trainee experiences of working in a new clinical workplace. Consultants are an essential resource that should be supported and developed to effectively provide this supervisory role within medical education and patient safety. The range of faculty development initiatives targeted at clinician-educators is growing [14]. These interventions primarily focus on teaching skills, assessment, instructional design, leadership, and educational scholarship [15]. A review of faculty development publications over the last 40 years concluded that there is a need to broaden the scope of faculty development beyond enhancing teaching effectiveness and expanding faculty development approaches to include workplace learning [14]. In the absence of formal guidance, consultants will likely continue to create their own ways of integrating new trainees into their clinical team and work environment. Fresh insights into how consultants currently manage the orientation of new trainees would be helpful for future faculty development in this area.

We undertook this constructivist grounded theory study to conceptualise the orientation of trainees starting work in a new clinical environment to support the continuing professional development of consultants.

## Methods

### Design

We used constructivist grounded theory [16] to explore the orientation of new trainees. With approval from our institutional Social Research Ethics Committee, potential participants were invited to a semi-structured individual interview. Email invitations were sent to Gastroenterology, Psychiatry, and Emergency Medicine consultants from three tertiary hospitals (*N* = 20). We chose these three specialties to include a diverse sample of participants to explore how the orientation of new trainees unfolds in various contexts. Potential participants were purposively selected to ensure equal distribution of participation between the chosen specialties and experience of supervising trainees in all grades of training. Fifteen consultants (five from each specialty) accepted the invitation and participated in the study. All those invited were consultants within Irish hospital settings and therefore had similar experiences and responsibilities of postgraduate medical training. Thus, there were no meaningful differences in characteristics between the participant group and those who declined. No further invitations were issued when data sufficiency was achieved. All participants who chose to participate in the study gave their written consent before the interview.

### Setting and context

In Ireland, all newly graduated doctors must complete a one-year internship consisting of four clinical rotations. After that, trainees may choose to specialise in a medical field, starting with 2-4 years’ basic specialist training (BST), followed by several more years in higher specialist training. A trainee on the General Internal Medicine BST undergoes eight rotations over two years, on the Emergency Medicine BST makes six rotations over three years, and on the Psychiatry BST goes through eight rotations over four years. In this paper, we use the term trainee for doctors who practice in postgraduate training posts. We refer to doctors who have completed specialist training and can practise independently as a specialist as consultants.

### Data collection and analysis

Study data were collected between February and December 2019. AW conducted an in-person semi-structured interview with each participant at their workplace. Interviews ranged between 60 and 120 min in length, were audio-recorded and transcribed verbatim. Data collection and analysis occurred concurrently. In compliance with grounded theory principles, the structure and content of the interview guide were modified throughout the study to reflect and sample for our developing theoretical insights [16]. This method guided theoretical sampling, which allowed us to proactively refine and test the emerging theory as new participants were interviewed. The interview questions prompted participants to reflect on, discuss, and explain their experiences of orientating new trainees to their clinical environment. Over time these questions were refined to explore the developing core concepts of the model more directly. Sampling and data collection were concluded once theoretical sufficiency appropriate for grounded theory methodology was reached, and the refined categories were rich enough to fully capture the analytic insights present within the data.

Consistent with the iterative process of constructivist grounded theory [17], interview transcripts were analysed using constant comparative techniques as soon as the data was collected to identify and refine recurring themes and the relationship between identified concepts. Following each instance of data acquisition and initial coding, emerging findings were presented, discussed, and refined at an analysis meeting between AW and DB. During these meetings, decisions were made about what were analytical priorities. A set of initial codes were collaboratively organised into focused codes which eventually contributed to the core categories of the theory. The focused codes were applied to a set of transcripts by AW. The results of focused coding were reviewed and discussed by the team, and opportunities to theoretically sample emerging insights were identified. Theoretical coding involved analysing the relationships between codes and connecting categories to provide a clear way of understanding the phenomenon we dealt with in this study. NVivo software [18] was used to facilitate data analysis. To further explore the resonance of the theoretical model we constructed from our data analysis, we presented the model at a medical education conference to fellow medical education researchers, medical educators (including consultant supervisors) and postgraduate trainees who had the opportunity to provide feedback on whether the results resonated with their own experiences.

### Reflexivity

As constructivists, we recognise that our backgrounds, experiences, beliefs, and interests shape our questions and interpretation of participants’ experiences. AW is a medical education researcher, physiotherapist, and lecturer in medical education responsible for undergraduate and postgraduate health professions’ education. DB is a medical education researcher, a physician and directs undergraduate and postgraduate health professions’ programmes. AW and DB supported each other’s reflexivity through frequent discussions of emerging concepts, questioning assumptions, and constantly comparing interpretations of the data throughout the research process.

## Results

**Fig. 1:**
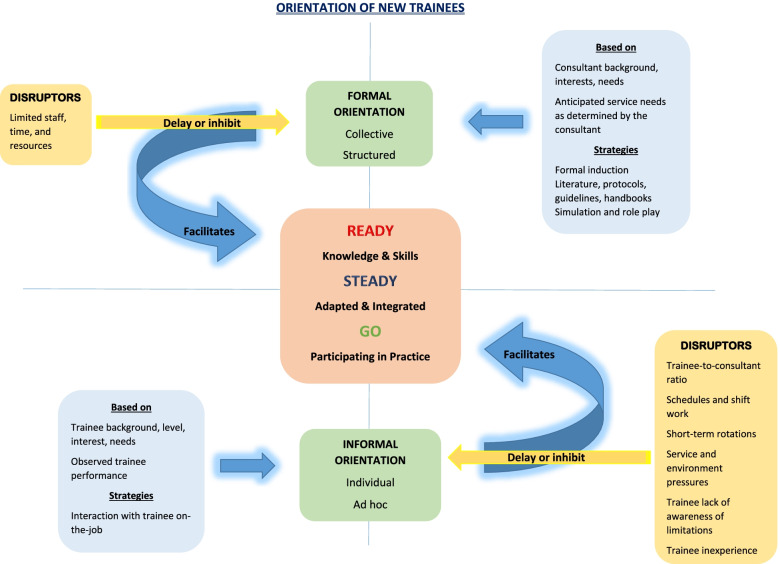
The ready-steady-go model of orientation of new trainees

A conceptual model of the strategies employed by consultants to orientate new trainees was constructed (Fig. 1). The core categories of the model and how they relate to each other are reported below, supported by participant quotes.

### Central category – ready, steady, go

Consultants’ central concern when introduced to a new cohort of trainees was that they had the required knowledge and skills (*ready*), were adapted and integrated into the new workplace and clinical team (*steady*), and safely participating in practice (*go*). Consultants were focussed on two main domains of quality service delivery - safety and efficiency – which were the main drivers for early interaction with new trainees. Ensuring that trainees were safe to practice was the highest priority for most participants: ‘*The first thing is to establish a safe level of delivery of care*’ (C2) and ‘t*o ensure that trainees are safe to practice in this environment*’ (C5). Efficient service delivery came a close second. There was a clear sense of urgency from consultants to get trainees to work at an acceptable rate: ‘*It’s a very busy job, and trainees need to be adaptable and need to be efficient’* (C4).

For trainees to practice safely and efficiently, consultants had to confirm they had the requisite knowledge and skills. The goal of trainee orientation also included integration and socialisation into the clinical team and ‘*to make trainees more comfortable with the environment*’ (C12). Consultants wanted trainees to be steady by feeling supported in their new location. Participants recognised that trainees had to be afforded a period of adaptation and that it was a ‘*steep learning curve*’ (C5) that required additional support during this time.

Orientation was characterised as a period of heightened intensity of interaction between consultants and new trainees. The ebb and flow of the intensity of supervision during the orientation phase were described as the following by a participant:

‘*After the changeover, we tend to supervise trainees more, and once we know that they are independent and capable, based on our direct supervision and conversation, formal and informal, we then know what sort of supervision they would require going forward*.’ (C7)

Trainee orientation did have an endpoint, was limited in duration and, according to this study’s participants, could range from one month: ‘*the first month would be the most important one because we need to establish what trainees are capable of*’ (C7) to six weeks: ‘*we would expect to have the measure of most of our trainees after about six weeks*’ (C12). The greatest indication that consultants had successfully integrated trainees was at the point when they knew trainees’ performance capabilities and that they could be trusted to deliver safe and efficient service. One participant considered orientation to be completed when:

*‘I had a good handle on their ability or lack of ability, their competency and whether I could trust them.’* (C14)

### Orientation strategies

Consultants employed two broad strategies: formal orientation and informal orientation. When both these strategies were used, they happened concurrently. The consultant-trainee interactions that occurred because of orientation strategies had temporal and inter-personal dimensions, which meant that trainee orientation alternated between collective or individual, structured or ad hoc, depending on the situation. Orientation strategies were also characterised by their shared goal of intensifying interaction between consultants and trainees to get trainees to a position where they were ready, adapted, integrated, and participating safely and efficiently in practice. In the words of one participant, these two strategies were summarised as follows:

*‘There is a collective piece, and then there is an individual piece in how we go about induction in psychiatry in our service… this provides lots of opportunities for trainees to learn, and it does ensure that there is a lot of contact between supervisors and trainees.’* (C11)

#### Formal orientation

Formal orientation was conducted collectively, meaning that new trainees were grouped and put through a similar set of experiences. Formal orientation was based on anticipated service requirements as determined by the consultants and their experiences of service provision in their place of work. Structured approaches were used in formal orientation, such as induction sessions. The use of literature, including guidelines, protocols and handbooks, was common, and sometimes tools such as simulation and role play. Looking at how participants described formal orientation, it became evident that this was a collective experience, which relied on standardised information and was driven by service needs particular to that workplace:

‘*In the first two weeks of their new rotation, we have an induction block…we generally group them together, we introduce them to the policies and the guidelines within the department and deliver detail on some of the very common presentations that we see.’* (C12)

The consultants wanted to cover a wide range of topics with planned orientation to provide trainees with a solid foundation to start and ‘*were very explicit about what is required of them from day one’* (C2). The participant then continued to explain what his goals were during planned initiation:

‘*Teaching the basic skill set for the most common problems presenting in this department and establishing criteria for when they need to call you… to educate the trainees in terms of their limitations, knowing their limitations, knowing absolutely when they need assistance, and then to educate them in terms of delivery of care. To do that, we did a two-week induction, and we had two training mornings a week for formalised teaching. Very early in the first two sessions, we tell them what we expect of them. We expect them to make decisions, and we expect them to see patients, and we expect them to ask if they don’t know.*’ (C2)

It was also important to provide trainees with information about the practicalities of daily service delivery and to set expectations regarding this at an early stage:

*‘With regard to my own patients, we will introduce them to the clinical care plan and how we would write it out and how you would manage patients who are admitted on to the ward. I will give them a copy of what I expect their note to look like, and I would show them how the blood tests are done.’* (C10)

Formal orientation involved mainly the verbal and written transfer of information, but in some instances, trainees were actively engaged through, for example, simulation activities so that the consultants could begin to get an idea of the new trainees’ level of knowledge and skills:

*‘We see trainees in action during the induction as well and see their level of engagement. The induction includes simulation, which gives us a chance to look at their technical skills.* (C5)

To round out formal orientation, administrative information was also disseminated during that time:

*‘Trainees would get a particular set of documents during induction around the job they are going into that sets out very clearly what their role is and would include contact details for those staff members when the relevant meetings are on, and their timetable.’* (C11)

#### Informal orientation

Informal orientation occurred at the level of the individual trainee with this type of orientation described as ‘*intense engagement with trainees*’ (C5). Consultants focused on individual trainee performance and their unique needs and interests:

‘*Each trainee is different and has different capacities and different interests and different abilities…. you have to get to know that with each of them when you’re on your own or if there are only the two of you*.’ (C15)

Informal orientation involved observation and interaction by the consultant with the trainee during service delivery. It was important for consultants to directly observe trainees performing procedures during orientation to check whether they could safely and efficiently do the work that was required of them:

‘*When it comes to endoscopic procedures, we do need to stand behind them in that early phase and especially for those who are in transition… that will tend to take place within the first four weeks and after that depending on how they have progressed they probably would require less supervision.’* (C7)

Feedback from the wider clinical team about individual trainee performance was another key informal orientation strategy. Feedback from senior doctors was important, but supervisors also considered the input from other allied health care professionals, in particular, nursing staff, who frequently interacted with new trainees:

‘*We have a cohort of doctors who were there before the changeover, and we would depend on them to feedback to us, and then we would make our own observations.*’ (C14)

Based on feedback information and in combination with their own observations, consultants tailored their orientation of trainees on an ad hoc basis.

### Disruptors of orientation

Formal and informal orientation did not always happen smoothly, and several disruptors were identified by participants that delayed and sometimes completely inhibited the orientation process. Disruptors specific to formal orientation were the lack of staff, time, and resources: *‘It’s a really busy service, and it can often happen that some of that formal induction doesn’t happen, and they get shoved right into the middle of it.’* (C10).

Disruptors specific to informal orientation were:

- A high trainee-to-consultant ratio
  - *‘We have twenty trainees and nine consultants… it’s hard if there is nine of you and then you’ve got twenty of them… that makes it difficult… there are fewer intimate relationships between the consultants and trainees.’* (C2)
- Schedules and shift work
  - *‘When they are working a week of nights and miss out on a whole week of days, they are getting less exposure, so sometimes it [orientation] can be a lot slower.’* (C4)
- The length of a rotation
  - *‘The relationship with a trainee at registrar level is a deeper relationship than that which you have with the interns and senior house officers which are in many cases fleeting or non-existent because they rotate through short intervals.’* (C7)
- Service and work environment pressures
  - *‘When it comes to working in an environment like this, that is as confronting as this one is, they can come apart. So, you’re watching out for that too.’* (C5)
  - *‘Some of these doctors may not be suited to the intensity of the environment, but we support them more, and we have a very close eye on them all of the time to make sure no one is struggling.’* (C1)
- Poor trainee recognition of their limitations
  - ‘*If they don’t come to me and ask questions, saying, for example, I know you told us how to do X, but can you just tell me again how to do that? If they don’t do that, they can’t be trusted. So, I need to pay greater attention to those. Those that are coming really regularly, I will feel more comfortable*.’ (C9)
- Limited trainee experience of the knowledge and skills required for service delivery in their new location
  - *‘Many trainees who come here have very little experience, so they feel out of their depth from the very beginning, and that’s not a comfortable place to be for anyone who is starting a new job*.’ (C8)

## Discussion

The conceptual model constructed from this analysis presents the key orientation strategies employed by consultants to facilitate trainees to be ready and supported to participate in practice in a new clinical workplace. This model suggests that a programme of collective, individual, formal, and informal interactions and experiences under supervision and attention to potential disruptors may be needed to integrate trainees successfully into a new clinical environment.

### Formal and informal orientation strategies

The orientation strategies at the core of this study involved both formal and informal elements. Formal orientation occurred in the context of organised induction and planned learning activities and aimed to generate explicit, structured knowledge and skills designed for the current workplace. Formal orientation happened within the clinical environment but not during service delivery. Yet, it was highly authentic and grounded in immediately transferable and relevant information. Previous research has similarly recognised the role of explicit supervisor-mediated teaching within the workplace curriculum and the availability of formal training and resources to new workers [19, 20].

Informal orientation involved individual interaction with and observation of a trainee by the consultant during day-to-day work. The information gathered during one-to-one engagement with the trainee and feedback from the broader clinical team shaped the structure and nature of informal orientation. Informal orientation was implicit, collaborative, and highly contextualised, all features of informal learning [20]. However, the focus on orientation differentiated this process from more general informal supervised workplace learning.

Both formal and informal orientation were planned and designed to heighten the intensity of engagement between new trainees and consultants. The intentionality of consultant engagement during orientation of a new trainee was directed towards the safety and efficiency of service delivery. These outcomes were achieved through purposeful facilitation of the orientation period so that trainees would be ready, steady, and going about the business of healthcare provision.

### Orientation and learning

The intentionality of consultant engagement during the orientation of new trainees was not explicitly aimed at learning, yet, mechanisms of supervised workplace learning [13] were key components of our findings: monitoring and entrustment. Previous research has suggested that these two mechanisms are supervisor driven and occur in the context of workplace learning [13] and that transitions are inseparable from learning and practice [2]. Therefore, it makes sense that these mechanisms of learning would appear in a study that drew on consultants’ experiences of integrating trainees into a new clinical workplace.

Monitoring involves both direct and indirect observation of trainees’ clinical performance [13]. Planned and direct observation has been previously identified as important during the initial phase of a supervisor-trainee relationship [21]. Direct observation, however, is not a perfect method for determining a trainee’s level of competence because of the impact the observer may have on the authenticity of a trainee’s performance [22]. Therefore, consultants must also depend on additional sources of information, such as feedback from other team members about a trainee’s clinical performance. As the examples of indirect observation found in this study, further research has shown that healthcare workers, such as nursing staff, support monitoring [23].

Entrustment is the mechanism through which trainees grow progressively independent as their competence develops [24, 25]. Participants in this study considered the orientation process to be completed when they developed trust in a trainee. Upon arrival in a new clinical workplace, a trainee’s status, skills, and responsibility are generally on a level footing between the old and the new. Trainee progress in terms of increased responsibility or complexity of work did not feature in this study. However, decreased consultant oversight was a sign of effective orientation.

### Disruptors to the supervision and orientation of new trainees

Like other socio-culturally constructed phenomena such as supervised workplace learning [13], orientation of new trainees was also enabled and constrained by a multitude of contextual factors. Several such restraining factors were identified in this study and were conceptualised as disruptors to orientation. These disruptors either delayed or completely inhibited orientation strategies.

One-to-one interaction between a consultant and a trainee during a working day was a critical component of informal orientation. Still, this time together was difficult to preserve when consultants’ and trainees’ schedules did not match. Research has shown that asynchronised, and fragmented working patterns can be detrimental to supervisor-trainee relationships [26] and learning and likewise have shown to be disruptive to orientation in this study. A high trainee-to-consultant ratio was also identified as a disruptor. It is difficult to pinpoint the root cause of this imbalance because workforce planning is complex and involves many moving parts. We know that the number of medical students graduating each year has never been greater [27]. Also, a shortage of consultants has been an acute problem for medical workforce systems and is predicted to worsen [28, 29]. In combination, these factors might suggest that there may not have been a proportionate increase of consultants to match the rise in the number of postgraduate trainees.

The impact that short-term rotations, and resulting discontinuity, have on learning, practice, and relationships has been well documented [3, 4, 12]. In this study, short rotations were disruptive to the orientation of new trainees also. Furthermore, the idea that our participants perceived inexperience and poor trainee recognition of their inexperience as disruptors reveals a difficult situation. Consultants would like trainees to be ready to go when they arrive so that service is fast, safe, and uninterrupted. The challenge is that learning in one context does not guarantee seamless integration into another. Invariably a period of adaptation must be tolerated and supported by consultants. This message urges us to reflect on current approaches to postgraduate medical training and the implications for service delivery and learning.

### Implications for practice and research

The findings of this study offer a way to raise the standards of postgraduate medical training and faculty development. Our model may be a valuable tool for supporting consultants’ reflective practice about how they supervise and integrate new trainees and the disruptors to these processes, which may arise in their workplace. The readiness of workplaces to afford orientation strategies play a critical part in the quality of the supervision of new trainees. A central feature of workplace learning is the affordances or the invitational qualities of workplaces [30]. Invitational qualities refer to the attributes of workplaces that invite individuals into their social practices [31]. As trainees try to find footing in a new workplace, its invitational qualities may be enhanced by actions such as planned formal and informal orientation strategies. The data from this study could be used to develop and increase the utility of available resources and protect consultants and trainees from the disruptors in clinical learning environments.

Individuals have specific abilities, previous experiences, and ways of making meaning of social situations (personal epistemologies) that shape how they engage with and learn through work activities and interactions [32]. These personal epistemologies may likewise shape trainees’ view of and engagement with orientation as they enter a new work environment. Trainees may not equally value invitations offered through orientation strategies. Therefore, enhancing our understanding would require exploring trainees’ perspectives on the quality of the invitations extended to them through the procedures described in this study. Knowing how orientation by consultants is perceived and engaged with by those it is intended for may provide valuable insights. Regarding methodology, a longitudinal research approach that moves with trainees through a series of rotations could give a more robust overview than a single moment in time inquiry. Future research in different hospital settings and countries would be helpful in refining theoretical insights further and contextualise new trainee orientation.

### Methodological strengths and limitations

Strengths of this study include the rich and diverse sample of consultants and strict observance of constructivist grounded theory principles [16]. Caution should be exercised about transferring the findings to different contexts without proper consideration for the influence of local culture and practice on the emergent properties of this model of orientation. Our focus was on the consultants’ perspective in this study, so trainees’ perspectives and experiences regarding the orientation strategies are unknown. However, future research to address this gap can now be strengthened by having this model available as a sensitising concept for data collection and analysis.

## Conclusions

Consultants use formal and informal strategies to orientate new trainees. Disruptors to these strategies were identified as valid priorities for improving trainee and consultant experiences in postgraduate medical education. The model of orientation constructed through this research could be a valuable tool to support faculty development initiatives, the reflective learning practice of clinical supervisors, and curriculum design. Future research should involve a longitudinal approach to explore trainee engagement with orientation upon entering a new clinical workplace.

## Data Availability

The data analysed in this study are available from the corresponding author on reasonable request, subject to ethical approval.

## Abbreviation

CGT: Constructivist grounded theory
